# Sentinel Nystagmus: The Key to Identifying Type II Oculocutaneous Albinism (OCA2) in the Pediatric Setting

**DOI:** 10.1155/crpe/5514931

**Published:** 2026-01-09

**Authors:** Janan Niknam, Arpineh Petrosyan, Victoria Agustin, Amanda Shoubaki

**Affiliations:** ^1^ College of Osteopathic Medicine, William Carey University, Hattiesburg, Mississippi, USA; ^2^ Department of Pediatrics, South Central Regional Medical Center, 1220 Jefferson St., Laurel, Mississippi, USA

**Keywords:** case report, nystagmus, oculocutaneous albinism

## Abstract

**Purpose:**

To present a case of type II oculocutaneous albinism (OCA2) diagnosed in infancy following the finding of nystagmus, and to review the diagnostic process and the management of this disorder.

**Observation:**

A 4‐month‐old female presented with subtle, roving eyes that were initially attributed to normal development. A subsequent evaluation by a pediatric ophthalmologist, prompted by a high index of suspicion, confirmed the findings of nystagmus, mild foveal hypoplasia, and astigmatism. Genetic testing confirmed the presence of pathological variants of the OCA2 gene, leading to a diagnosis of oculocutaneous albinism.

**Conclusion and Importance:**

This case highlights the importance of a meticulous ophthalmologic examination and a high index of suspicion in pediatric care. The early finding of nystagmus can be the key to a timely diagnosis of OCA2. This allows for early intervention to optimize visual development and allows for multidisciplinary management and genetic counseling for the family. This case underscores the need for ongoing education in enhancing the early detection of OCA.

## 1. Introduction

Oculocutaneous albinism (OCA) is an autosomal recessive disorder, impacting the presence of melanin pigment in the skin, hair or eyes [1]. Reports regarding the prevalence of OCA vary depending on the region, with a reported prevalence of 1 in 14,000 worldwide [1]. Various genes have been implicated in the development of OCA [1]. To date, eight genes causing OCA have been identified, OCA 1–8 [1]. The most common gene mutation causing OCA varies by region, with the OCA1 gene being the most commonly affected in Caucasians, responsible for approximately 50% of cases worldwide. The OCA2 subtype is the most common subtype in Africa [2]. The OCA2 gene mutation results in an abnormal membrane protein responsible for melanogenesis [3].

Various ocular manifestations of OCA have previously been described. Hypopigmentation in the eyes results in reduced visual acuity primarily due to foveal hypoplasia and misrouting of optic nerve fibers [3]. The degree of visual impairment is largely dependent on the degree of pigment production, with those with some degree of residual pigment production having better visual acuity outcomes [3]. We present the case of a 4‐month‐old female with a finding of nystagmus at a routine pediatrician visit, prompting an ophthalmology consult, with a subsequent genetic confirmation of an OCA2 mutation. This case is unique as the diagnosis was prompted by a subtle finding of nystagmus during a routine physical exam, initially misattributed to normal development, underscoring the critical need for a high index of suspicion.

## 2. Case Presentation

The patient is a 4‐month and 3‐week‐old female presenting to the pediatric clinic due to parental concerns about an ear infection. She was born at 38‐week gestation to an 18‐year‐old G1P0 mother. Prenatal complications included hyperemesis gravidarum and oligohydramnios. The patient had a normal newborn course. The patient had been tugging on both ears for the past two days. The parents denied fever, rashes, nausea, or vomiting. However, the child had had 1‐2 episodes of diarrhea, potentially attributed to teething by her mother. The patient has fair skin and hair. On physical exam, her vitals were as follows: temperature: 36.5°C, heart rate: 146 bpm, respiratory rate: 48, and SpO_2_: 98% on RA. The physical exam showed roving eyes, with a diagnosis of nystagmus, as well as a bulging anterior fontanelle. The remainder of the physical exam was unremarkable. The patient was referred to the hospital for a CT scan of the head without contrast and an ophthalmology consultation.

The CT scan of the head showed benign enlargement of the subarachnoid space and confirmed the absence of hydrocephalus or any acute intracranial abnormalities, suggesting benign causes for the bulging anterior fontanelle. The patient was also seen by an ophthalmologist. The exam showed very occasional horizontal bilateral nystagmus‐like beats. The patient’s eyes could fixate on an object but would tend to drift to the left or right. The remainder of the ophthalmological exam was unremarkable. This exam was documented as “normal for developmental age” with the absence of light sensitivity or true nystagmus. The patient was instructed to follow up with a pediatric ophthalmologist.

At outpatient follow‐up, the pediatric ophthalmologist noted the presence of nystagmus, the absence of iris transillumination, and the presence of mild foveal hypoplasia with a possible variant of OCA. The patient was instructed to continue following up with the pediatric ophthalmologist every six months or sooner if problems arise. The purpose of follow‐up appointments was to ensure proper visual development and to monitor for problems such as strabismus and amblyopia. The patient’s visual acuity measured at 2 years of age showed a visual acuity of 20/80 OU, further necessitating corrective lenses to ensure proper visual development. At 2 years and 9 months old, the patient was found to have persistent nystagmus, foveal hypoplasia, and astigmatism in both eyes. Additionally, the patient was noted to have a left‐sided head tilt, compensating for the nystagmus. The prospect of a surgical correction for torticollis was discussed. However, the family declined surgery. The patient had been wearing glasses for vision correction, and a subsequent genetic test was performed to check for the presence of OCA. The genetic testing confirmed an autosomal recessive mutation in the OCA2 gene, thereby confirming the diagnosis of OCA. Genetic testing revealed two pathologic variants of the OCA2 gene, the c.1327 G > A p (V443I) and the c.632 C > T p. (P211L). Genetic counseling regarding the implications of these results was provided to the family. At the 3‐year wellness visit, the patient was doing well and meeting appropriate developmental milestones. The most recent visual acuity was measured at 20/60 OU at the age of 3 years. The child continues to wear corrective lenses for vision.

## 3. Discussion

OCA comprises defective pigment production affecting the skin, hair, and eyes [4]. The degree of residual pigmentation varies depending on the subtype of OCA, with a greater degree of pigment present in certain subtypes, such as the OCA2 subtype. The increased availability of genetic sequencing has allowed for the diagnosis of OCA in patients with an unclear clinical picture [5]. Early diagnosis is important, particularly in the pediatric population, to facilitate timely genetic counseling for the family, optimize visual acuity through early intervention, and ensure parents are equipped with the knowledge to mitigate long‐term risks associated with OCA. Foveal hypoplasia is an important early sign of OCA. However, the normal presence of an immature fovea at birth can lead to a delayed diagnosis of OCA [6]. Several findings suggestive of OCA have been reported in the literature. Iris transillumination is a reliable finding in patients with OCA and can be easily checked at routine pediatrician visits [6].

Various clinically important manifestations can be seen in individuals with OCA. For instance, due to the role of skin melanin in protection against ultraviolet radiation from the sun, individuals with OCA have a higher risk of skin cancer development, further necessitating prompt diagnosis and intervention to mitigate the risk of malignancy [7]. In addition to the skin findings, various ocular manifestations of OCA have been reported. These manifestations include a nonprogressive decline in visual acuity, strabismus, nystagmus, foveal hypoplasia, and abnormal decussation of retinal ganglion cells in the optic chiasm [8]. The mainstay of treatment for OCA is strategies to improve the quality of life with measures such as refractive lenses and eye muscle surgeries to improve the individual’s visual outcomes and the use of effective sun protection strategies to decrease skin cancer risk [7, 8]. These measures are important in the pediatric population to ensure optimal brain and visual development later in life. Guidelines have been described in the literature regarding the use of corrective lenses for children with OCA. In children over the age of two with albinism, corrective lenses are required with refractive errors > 2.50 D for myopia, > 2.00 D for hyperopia, and > 2.00 D for astigmatism [6]. Protective measures against skin cancer are recommended to begin as early as possible to reduce the lifetime risk of skin malignancies.

This case demonstrates the importance of attentiveness to subtle ophthalmologic symptoms such as roving eyes or nystagmus and prompt intervention and further evaluation. Infantile nystagmus is rarely benign and mandates a thorough ophthalmologic and neurologic workup. In a study of 202 children with infantile nystagmus, Bertsch et al. demonstrated an identifiable underlying etiology in 90% of patients. In this study, the most common cause was found to be OCA, accounting for 19% of the cohort, followed by Leber Congenital Amaurosis (LCA) and non‐LCA retinal dystrophy. These findings emphasize the importance of maintaining a high index of suspicion and conducting a thorough neurologic and ophthalmologic workup for pediatric patients with nystagmus [9]. A high index of suspicion and referral to a pediatric ophthalmologist is crucial, as evident in this case with a normal initial ophthalmologic exam. Prompt diagnosis is important to optimize the child’s visual development and to address potential compensatory changes, such as torticollis in this case. Timely genetic diagnosis also allows for genetic counseling for the family, as well as counseling regarding skin cancer risks and sun protection.

## 4. Conclusion

Early diagnosis of OCA in the pediatric population is crucial to ensure proper development and to protect the child against environmental factors to mitigate the risk of skin malignancies. The dianogis of OCA can be challenging, especially in cases where the presenting symptoms can be subtle. Genetic testing can be helpful in confirming the diagnosis of OCA in patients presenting with ophthalmic manifestations suspicious of OCA. The diagnosis of OCA in a pediatric patient should be followed up with regular ophthalmologic exams as well as dermatologic exams to ensure proper visual development and reduce the risk of skin cancer development.

## Consent

Written informed consent was obtained from the patient’s mother for the publication of this case report.

## Conflicts of Interest

The authors declare no conflicts of interest.

## Author Contributions

All authors attest that they meet the current ICMJE criteria for Authorship.

## Funding

No funding or grant support was obtained for this study.

## Data Availability

Data sharing is not applicable to this article as no datasets were generated or analyzed during the current study.
